# A single oral dose of Silodosin and Diclofenac sodium is effective in reducing pain after ureteric stent removal: a prospective, randomized, double blind placebo-controlled study

**DOI:** 10.1186/s40064-015-1662-7

**Published:** 2016-01-07

**Authors:** Goto Gangkak, Ram Dayal Teli, Sher Singh Yadav, Vinay Tomar, Shivam Priyadarshi, Satinder Pal Aggarwal

**Affiliations:** Department of Urology and Renal Transplantation, Sawai Man Singh Medical College and Hospital, Jaipur, Rajasthan India

**Keywords:** Silodosin, Diclofenac sodium, Pain, Stent removal, Visual analogue score, Ureteral stone, Renal stone

## Abstract

A prospective double-blinded placebo controlled randomized study was conducted in our institute to compare the efficacy of oral Silodosin, an alpha 1 selective antagonist and Diclofenac in relieving pain after stent removal. All patients with unilateral stent placement following renal and ureteric stone endoscopic surgery were randomized into four groups: group A (Placebo), group B (Diclofenac), group C (Silodosin) and group D (combination of Diclofenac and Silodosin). Visual analogue score (VAS score) and other relevant parameters were assessed during OPD visit. Patients were handed over randomized drug envelope and asked to take the medications one hour before the stent removal. Patients were contacted 24 h after stent removal and relevant parameters were recorded. The mean VAS scores were significantly decreased in Diclofenac (2.9), Silodosin (3.08) and combination group (2.85) when compared to placebo (4.20) (p < 0.001). However there was no statistically significant difference in VAS scores between the treatment groups, i.e., group B, C and D (p > 0.5). Analgesics requirement and severe pain rates were not significantly reduced (p = 0.07, 0.35) in the three treatment groups when compared to placebo. Thus Silodosin and Diclofenac, both are effective in preventing pain after stent removal.

## Background

Ureteral stents have become an indispensable tool for the urologist but they are often associated with significant morbidity. A significant number of patients often report pain and urinary symptoms with indwelling stents (Joshi et al. 2003). Extensive studies have been done to assess stent-related discomfort but these studies have primarily assessed the stent-related pain, while stent was in situ (Dellis et al. 2010; Gupta et al. 2010; Beddingfield et al. 2009; Rane et al. 2001; Deliveliotis et al. 2006; Damiano et al. 2008). But it is a common observation that many patients complain of renal colic type of pain after stent removal, which often requires admission and additional analgesics. There has been only one previous study, describing this phenomenon of pain after stent removal by Tadros et al. (2012) and they concluded that significant number of patients had pain after stent removal and Rofecoxib, a selective cyclooxygenase (COX) inhibitor was effective in reducing pain after stent removal. They ascribed the pain relief to the analgesic, anti-inflammatory as well as smooth muscle relaxing properties of the non-steroidal anti-inflammatory drug (NSAID). Diclofenac, a long acting NSAID with a quick onset of action has been quite effective in treatment of renal colic (Davies and Anderson 1997; Tankó and Tamás 1995–1996; Hurault and Ryckelynck 1989). Alpha blockers have been found to be quite effective in relieving stent-related pain (Deliveliotis et al. 2006; Damiano et al. 2008). Silodosin is a long acting alpha 1 selective antagonist with rapid onset of action (Michel 2010). But no study is available to assess their effectiveness in relieving pain after stent removal. So, we conducted this study to compare the effectiveness of oral Silodosin, Diclofenac and their combination in reducing pain after stent removal.

## Methods

The prospective, randomized, double-blinded, placebo-controlled study was conducted in our department after taking clearance from our institute’s ethics committee. All patients above 17 years and below 55 years undergoing unilateral ureteral stenting following renal and ureteric stone surgery were included. In all the patients, 5 Fr double J (DJ) polyurethane ureteral stent was used. DJ stent was kept for a period of 3 weeks before removal. A total of 272 patients were enrolled in the study from January 2014 to March 2015. Patients with history of peptic ulcer disease, liver impairment, chronic renal failure, coronary artery disease, bleeding diathesis, asthma, urinary tract infections (UTI), chronic painful conditions like arthritis, pregnancy, allergy to medications, significant lower urinary tract symptoms (LUTS) and use of alpha blockers and residual calculus were excluded. Patients with complications during stent removal like hematuria and mucosal injury were also excluded. Out of 272 patients, 240 patients were included in our study after excluding 32 patients not fulfilling the inclusion criteria. The patients were then randomized into four groups: group A (Placebo), group B (Diclofenac), group C (Silodosin) and group D (combination of Diclofenac and Silodosin) using a computer generated model. 60 patients were taken in each group. A vitamin tablet containing folic acid was used as placebo. 50 mg Diclofenac sodium oral tablet and 8 mg Silodosin oral tablet were used in the treatment groups. All medications were placed in numbered envelope as per the computer generated model. All patients and investigators were blinded to the medicine identity and randomistaion design till the end of the study. Patients were handed over the next numbered envelope on their OPD visit and Visual analogue score (VAS score) was also recorded at the same time. Visual analogue score was taken on a scale from zero to ten, zero meaning no pain to 10 meaning excruciating pain. Patients were asked to take the medications 1 h before the procedure. All patients received a single dose of levofloxacin 500 mg before stent removal as per our department protocol. The surgeon removing the stent was also blinded about the grouping. Stent removal was performed under local anesthesia using 2 % xylocaine jelly under vision with 8/9.8 ureteroscope. All patients were contacted after 24 h and VAS score, additional medications requirement and site of pain and any other relevant parameters were recorded. Additional analgesics dose was measured in mgs of i.v morphine equivalents. Patients with severe pain or patient willing for admission were readmitted.

## Statistics

Chi square test was used for sex-data analysis. One way Anova, Mann–whitney test and Kruskal–Wallis test were used for analyzing all the other datas. Our sample size calculation of 50 patients per study arm was powered to detect a 20 % decrease in the VAS score with a 95 % confidence level.

## Results

**Fig. 1:**
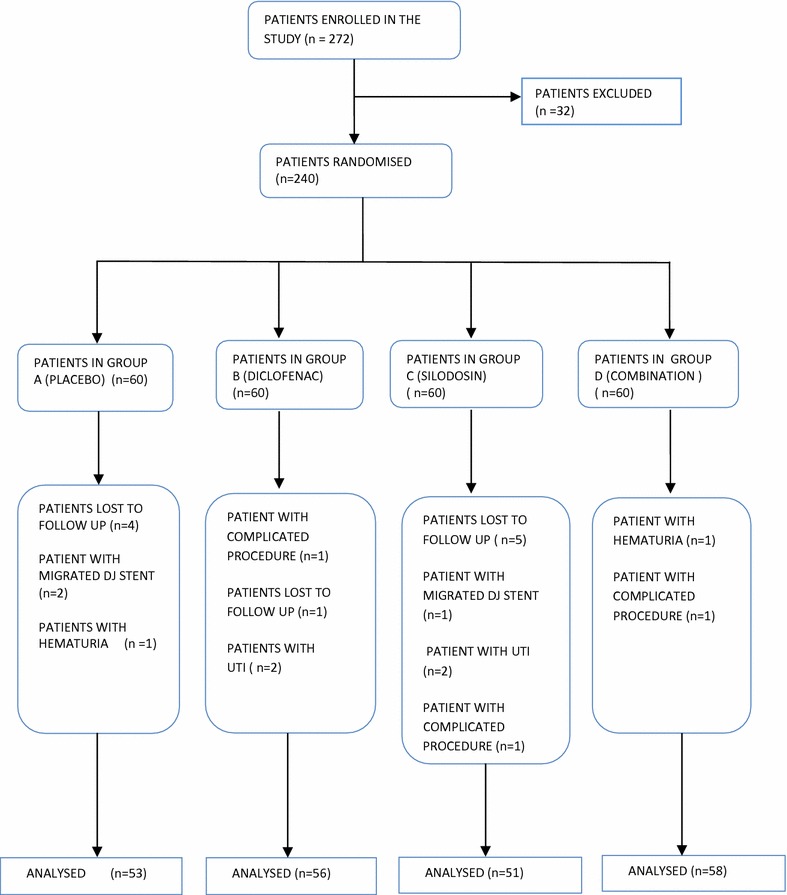
The flowchart showing the study design

Out of 240 patients included in the study, 22 patients were further excluded from final analysis (Fig. 1). 10 patients did not report back to us, 3 patients had migrated stent, 3 patients had complicated procedure, 2 patients had significant hematuria following stent removal and 4 patients had febrile UTI requiring intravenous (i.v) antibiotics and admission. So a total of 218 patients were taken up for analysis.

All the four groups were analysed for baseline characteristics for comparability (Table 1). The results were comparable for all the assessed baseline parameters. The age of patients ranged from 19 to 55 years in our study and all groups were comparable for age (p = 0.17). Number of male and female patients were not equally distributed in all the four groups but there was no statistically significant difference (p = 0.38). Diclofenac group had more number of male patients and less female patients, but it was statistically insignificant (p = 0.23). Most common diagnosis was ureteric stone (89 %). There was no difference in diagnosis among various groups (p = 0.45). One hundred and nineteen patients had stents on the right side and 99 patients had on the left side. There was no difference between the side of stenting among all groups (p = 0.90). Duration of stenting also was found to be statistically insignificant among all groups (p = 0.08). The mean duration of stent insertion was 23.5, 25.4, 21.7 and 22.5 days in group A, group B, group C and group D, respectively. The mean pre-stent removal VAS score was 3.4, 3.2, 3.7 and 3.1 in group A, group B, group C and group D, respectively (p = 0.98).

**Table 1:** Demographics and outcome of the patients

Variables	Placebo	Diclofenac	Silodosin	Silodosin + Diclofenac	p value
	Group A	Group B	Group C	Group D	
Mean ± SD age in years	34.7 ± 10.8	36.4 ± 13.5	32.3 ± 9.9	40.2 ± 9.8	0.17
Sex M/F	40/13	45/11	36/15	42/16	0.38
Diagnosis					
Ureteric stone	47	51	48	49	0.26
Renal stone	6	5	3	9	
Side					
Left	28	18	21	31	0.90
Right	25	38	30	27	
Mean ± SD duration of stenting in days	23.5 ± 3.7	25.4 ± 2.6	21.7 ± 2.3	22.5 ± 2.8	0.06
					A–C (0.01)
Mean ± SD pre-stent removal VAS score	3.4 ± 0.9	3.5 ± 0.9	3.7 ± 1.0	3.2 ± 0.8	0.98
Mean ± SD post stent removal VAS score	4.2 ± 1.3	2.9 ± 1.1	3.1 ± 1.2	2.8 ± 1.0	<0.001 B–A (<0.001) C–A (<0.001) D–A (<0.001)
Post stent removal VAS score					
≤5	44	53	46	57	0.35
>5	9	3	5	2	
Mean ± SD additional analgesics requirement in mg (i.v morphine equivalents)	9 ± 0.7	4 ± 0.9	6 ± 0.8	3 ± 1.1	0.07

**Fig. 2:**
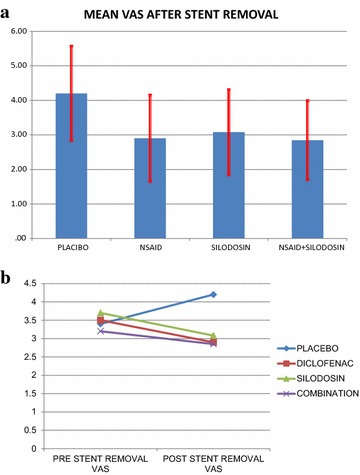
**a** The mean post stent removal VAS score in all the groups. **b** The change in VAS score between various groups

All the four groups were analysed for post stent removal VAS score. The mean post stent removal VAS score measured at 24 h were 4.2 in placebo group, 2.9 in Diclofenac group, 3.08 in Silodosin group and 2.85 in the combination group (Fig. 2a, b). The post stent removal VAS score was significantly lower in Diclofenac group as compared to Placebo group (p < 0.001). Similar results were obtained for Silodosin vs Placebo group (p < 0.001). But there was no statistical difference between the Diclofenac and Silodosin group (p = 0.30). The combination group (group D) had significant reduction in VAS score as compared to placebo (p < 0.001) but there was no significant difference when compared to Silodosin (p = 0.30) and Diclofenac (0.95). 9 patients (17 %) in Placebo group reported to have severe pain, defined as VAS score >5, but the incidence was not statistically significant when compared to all the three treatment groups (p = 0.08).

Loin pain was the most common presentation after stent removal, however it occurred with similar incidence in all three groups (p = 0.32). Only 5 % of the patients experienced suprapubic pain in Silodosin group but it was not statistically significant as compared to all other groups (p = 0.15). The mean additional analgesic requirement was least in combination group (3 mg), but did not achieve statistical significance (p = 0.70). The readmission rate also was similar in all four groups. No treatment-related significant side effects were seen in any of the groups.

## Discussion

Ureteral stents are associated with significant pain and discomfort (Joshi et al. 2002, 2003; Pollard and Macfarlane 1988). Joshi et al. reported that 80 % of patients experienced stent-related pain (Joshi et al. 2003). Numerous studies have been done to reduce stent-related pain and discomfort using drugs like alpha blockers, anticholinergics, phosphodiesterase inhibitors etc., new stent designs, stent materials and stent dimensions (Dellis et al. 2010; Gupta et al. 2010; Beddingfield et al. 2009; Rane et al. 2001; Deliveliotis et al. 2006; Damiano et al. 2008). Most of the available literature focuses on the stent-related morbidity when stent was in situ. But to any urologist, it is not unusual to have patients complaining of renal colic like pain after stent removal which often require additional analgesics and admission due to the severity of pain. Pain during stent removal probably occurs due to activation of nociceptors, the friction between stent and mucosa leading to ureteral smooth muscle irritability, trigonal irritation as well as pressure-induced changes in pelvi-calyceal system. However, this phenomenon of pain after stent removal has remained neglected and unreported. Tadros et al. (2012) first studied the efficacy of Rofecoxib in relieving pain after stent removal in a placebo-controlled randomized study and found that a significant number of patients complained of severe pain after stent removal in the placebo group and none in the NSAID group (8). NSAIDs are considered the gold standard drugs for renal colic (Davies and Anderson 1997; Tankó and Tamás 1995–1996; Hurault and Ryckelynck 1989). Numerous animal studies as well human studies have shown that NSAIDs cause significant decrease in ureteral contractility as well as decrease in renal blood flow (Chaignat et al. 2008; Perlmutter et al. 1993). Chaignat et al. (2008) have also demonstrated the presence of COX-1 and COX-2 receptors in human ureters. Nakada et al. (2000) reported that selective COX inhibitors and non-selective COX inhibitor led to significant reduction in ureteral contractility in both porcine and human ureteral tissues. Also, alpha blockers have been found to be effective in relieving stent-related pain (Deliveliotis et al. 2006; Damiano et al. 2008). Alpha 1 receptors have been found to be widely distributed in human ureter and bladder (Michel and Vrydag 2006; Itoh et al. 2007). Decrease in ureteral contractions as well as trigonal irritation are the likely mechanisms for the relief of stent-related pain with alpha blockers. Beddingfield et al. (2009) also reported a significant reduction in stent-related pain as well as reduction in narcotic dose with the use of Alfuzosin.

In our study, oral Diclofenac, which has a rapid onset of action and long t½ in inflamed tissues (Davies and Anderson 1997; Tankó and Tamás 1995–1996; Hurault and Ryckelynck 1989), significantly reduced pain after stent removal as compared to placebo (p ≤ 0.001). There was also less incidence of severe pain and additional analgesics requirement when compared to placebo but it was not statistically significant (p = 0.35, 0.56). Thus, the pain relief observed with Diclofenac was probably due to its inhibitory actions on ureteral contractility and renal blood flow as well as its analgesic and anti-inflammatory properties. However, the inflammation and edema may takes days to subside. So, this action probably plays a less significant role in the described setting.

In our study, there was significant reduction in pain in the Silodosin group as compared to placebo (p ≤ 0.001). Patients had less severe pain in Silodosin group as compared to placebo group but it was not statistically significant (p = 0.9). The mechanism of pain relief described in previous studies probably explains the pain relief seen with Silodosin in our study. The ureteral dilatory effect as well the decrease in ureteral and detrusor contractility by Silodosin are the likely mechanisms responsible for the pain reduction observed with Silodosin. There was also less incidence of suprapubic pain in Silodosin group but the results were not statistically significant (p = 0.15). This is probably related to Silodosin’s similar effect on lower urinary tract symptoms (LUTS) in BPH patients.

There was no difference in pain relief between both Silodosin and Diclofenac groups (p = 0.48). There was higher pain reduction seen with combination of Diclofenac and Silodosin but it did not attain statistical significance (p = 0.9, 0.40). Possible explanation is that there is probably certain overlap of different mechanisms of pain reduction involved between both the drugs. however The incidence of severe pain (VAS > 5) was also least in the combination group (3.4 %) when compared to all the other groups, but contrary to previous study, it was not statistically significant (p = 0.10). Tadros et al. demonstrated significant reduction in analgesic requirement with NSAIDs. However, the additional analgesics requirement in our study was least in combination arm but it was not statistically significant (p = 0.70). However we did not keep any objective criteria for use of additional analgesia and analgesics are used on demand basis. So it is likely that these factors might have influenced on our result.

Use of both Diclofenac and Silodosin and their combination did not result in any significant side effects in our study. Like other non-steroidal anti-inflammatory drugs, gastrointestinal side effects are the most commonly reported adverse effects of Diclofenac and seen in about 10 % of patients. These side effects have been found to be dose related (Lehtola and Sipponen 1977). These side effects with Diclofenac dose of 75–125 mg have been much lower than with aspirin 3–5 g (Brogden et al. 1980). Silodosin is comparatively a much safer drug with excellent side effect profile due to its unmatched uro-selectivity (Van Dijk et al. 2006). Silodosin has been reported to be frequently associated with abnormal ejaculation however, its clinical relevance remains unclear, as subjects rarely discontinue treatment due to this side effect (Marks et al. 2009). Moreover, with the single dosing of both the drugs, these side effects are unlikely to be of clinical significance, which is supported by our study results.

Thus our study supports the use of Diclofenac and Silodosin in reducing the pain after stent removal. However, ours is a preliminary study and further research is required in this field to better understand the phenomenon of pain after stent removal and the mechanisms of pain reduction involved.

However, there are certain shortcomings to our study. We took the pain score only after 24 h. If we could have taken more frequent pain scores before and even after 24 h, we could have better demonstrated and understood the pain dynamics involved. Moreover, with studies with a larger sample size more clear conclusions could have been derived.

## Conclusion

We found that use of single oral dose of Diclofenac and Silodosin is very effective in relieving pain after stent removal and the combination was not found to be more effective than the single agent. Thus, we recommend use of oral Diclofenac or Silodosin before stent removal to prevent pain after stent removal.

## References

[CR1] Beddingfield R, Pedro RN, Hinck B, Kreidberg C, Feia K, Monga M (2009). Alfuzosin to relieve ureteral stent discomfort: a prospective, randomized, placebo controlled study. J Urol.

[CR2] Brogden RN, Heel RC, Pakes GE, Speight TM, Avery GS (1980). Diclofenac sodium: a review of its pharmacological properties and therapeutic use in rheumatic diseases and pain of varying origin. Drugs.

[CR3] Chaignat V, Danuser H, Stoffel MH, Z’brun S, Studer UE, Mevissen M (2008). Effects of a non-selective COX inhibitor and selective COX-2 inhibitors on contractility of human and porcine ureters in vitro and in vivo. Br J Pharmacol.

[CR4] Damiano R, Autorino R, de Sio M, Giacobbe A, Palumbo IM, D’Armiento M (2008). Effect of tamsulosin in preventing ureteral stent-related morbidity: a prospective study. J Endourol.

[CR5] Davies NM, Anderson KE (1997). Clin Pharmacokinet.

[CR6] Deliveliotis C, Chrisofos M, Gougousis E, Papatsoris A, Dellis A, Varkarakis IM (2006). Is there a role for alpha1-blockers in treating double-J stent related symptoms?. Urology.

[CR7] Dellis A, Joshi HB, Timoney AG, Keeley FX (2010). Relief of stent related symptoms: review of engineering and pharmacological solutions. J Urol.

[CR8] Gupta M, Patel T, Xavier K (2010). Prospective randomized evaluation of periureteral botulinum toxin type A injection for ureteral stent pain reduction. J Urol.

[CR9] Hurault LB, Ryckelynck JP (1989). The role of non steroidal anti-inflammatory agents in the treatment of renal colic. Nephrologie.

[CR10] Itoh Y, Kojima Y, Yasui T, Tozawa K, Sasaki S, Kohri K (2007). Examination of alpha 1 adrenoceptor subtypes in the human ureter. Int J Urol.

[CR11] Joshi HB, Okeke A, Newns N, Keeley FX, Timoney AG (2002). Characterization of urinary symptoms in patients with ureteral stents. Urology.

[CR12] Joshi HB, Stainthorpe A, MacDonagh RP, Keeley FX, Timoney AG, Barry MJ (2003). Indwelling ureteral stents: evaluation of symptoms, quality of life and utility. J Urol.

[CR13] Lehtola J, Sipponen PA (1977). A gastroscopic and histological double-blind study of the effects of diclofenac sodium and naproxen on the human gastric mucosa. Scand J Rheumatol.

[CR14] Marks LS, Gittelman MC, Hill LA, Volinn W, Hoel G (2009). Rapid efficacy of the highly selective α1A-adrenoceptor antagonist Silodosin in men with signs and symptoms of benign prostatic hyperplasia: pooled results of 2 phase 3 studies. J Urol.

[CR15] Michel MC (2010). The Pharmacological Profile of the a1A-Adrenoceptor Antagonist Silodosin. Eur Urol Suppl.

[CR16] Michel MC, Vrydag W (2006). Alpha1-, alpha2- and beta-adrenoceptors in the urinary bladder, urethra and prostate. Br J Pharmacol.

[CR17] Nakada SY, Jerde TJ, Bjorling DE, Saban R (2000). Selective cyclooxygense-2 inhibitors reduce ureteral contraction in vitro: a better alternative for renal colic?. J Urol.

[CR18] Perlmutter A, Miller L, Trimble LA, Marion DN, Vaughan EDJ, Felson D (1993). Toradol, an NSAID used for renal colic, decreases renal perfusion and ureteral pressure in a canine model of unilateral ureteral obstruction. J Urol.

[CR19] Pollard SG, Macfarlane R (1988). Symptoms arising from double-J ureteral stents. J Urol.

[CR20] Rane A, Saleemi A, Cahill D, Sriprasad S, Shrotri N, Tiptaft R (2001). Have stent-related symptoms anything to dowith placement technique?. J Endourol.

[CR21] Tadros NN, Bland L, Legg E, Olyaei A, Conlin MJ (2012). A single dose of a non-steroidal anti-inflammatory drug (NSAID) prevents severe pain after ureteric stent removal: a prospective, randomised, double-blind, placebo-controlled trial. BJU Int.

[CR22] Tankó A, Tamás G (1995–1996) The use of Voltaren (diclofenac sodium, Ciba) in acute renal colic. Acta Chir Hung 35(3–4):285–290 9262725

[CR23] Van Dijk MM, de la Rosette JJMCH, Michel MC (2006). Effects of a1-adrenoceptor antagonists on male sexual function. Drugs.

